# Prominent features of platelet count, plateletcrit, mean platelet volume and platelet distribution width in pulmonary tuberculosis

**DOI:** 10.1186/2049-6958-7-38

**Published:** 2012-10-31

**Authors:** Füsun Şahin, Esra Yazar, Pınar Yıldız

**Affiliations:** 1 Department of Pulmonology, Yedikule Chest Diseases and Thoracic Surgery Training and Research Hospital, Zeytinburnu/İstanbul, Postal Code: 34760, Turkey

**Keywords:** Mean platelet volume, Platelet, Plateletcrit, Platelet distribution width, Pneumonia, Pulmonary tuberculosis, Thrombocytosis

## Abstract

**Background:**

We aimed to investigate the relation of platelet count (PLT) and plateletcrit (PCT), mean platelet volume (MPV) and platelet distribution width (PDW) with other acute phase reactants and radiological extent in pulmonary tuberculosis (PTB).

**Methods:**

One hundred patients with PTB (Group 1), 50 patients with community-acquired pneumonia (Group 2) and 28 healthy control individuals (Group 3) were included in this analytic study.

**Results:**

WBC (White Blood Cell), ESR (Eritrocyte Sedimentation Rate), CRP (C-Reactive Protein), PLT and PCT values were both in Group 1 and Group 2 than in Group 3. PDW values were significantly higher in Group 1 than Group 3. WBC, ESR and CRP values were lower, while PLT and PCT values were higher in the Group 1 compared to Group 2 (p < 0.001). PLT was positively correlated with CRP and ESR values in the tuberculosis group (p < 0.001), while it was not correlated with CRP and ESR in the pneumonia group (p > 0.05). ESR, CRP, PLT and PCT values were found higher in radiological advanced stage (Stage 3) patients with PTB, while hemoglobin (Hb) was found lower (p < 0.05). Higher WBC, ESR, CRP and PCT values as well as radiological advanced stage were more common in PTB patients with thrombocytosis compared to the patients with normal platelet count, whereas Hb was found lower in these patients.

**Conclusions:**

This study indicates that reactive thrombocytosis and higher PCT and PDW develop frequently in PTB and there is a relation between thrombocytosis and acute phase reactants, that is the inflammatory response. In addition, tuberculosis with radiological advanced stage is seen more frequently in the patients with thrombocytosis and higher PCT, drawing attention to the possible role of platelets in the cell-based immune process of tuberculosis.

## Background

Besides hemostasis, platelets are accepted to play a role also in inflammatory response [1-3]. It has been demonstrated that changes in platelet count, especially during the course of bacterial infections might be associated with the severity and mortality of the infection. Moreover, patients with thrombocytosis (among the patients hospitalized due to community-acquired pneumonia) have been reported to carry a high risk of mortality [3]. Existence of thrombocytosis has been shown in many studies investigating the hematological changes in tuberculosis [2,4-6]. Furthermore, the increase in the platelet count has been reported to be correlated with the severity of tuberculosis and acute phase reactants [1,2]. Thus, in addition to clinical and radiological findings, it has been indicated that platelet count and indices might be used together with the other acute phase reactants in order to define activation of tuberculosis [1,2]. Objective of this study was to investigate the relation between the existence of thrombocytosis, platelet and indices with the other acute phase reactants and the radiological stage of the disease in patients with PTB. In addition, patients who developed acute bacterial pneumonia due to non-tuberculous factors were also included in the study in order to discuss the possible role of platelets in immunopathogenesis of PTB.

## Methods

This is an analytic study. The study was performed in accordance with the principles of the Declaration of Helsinki and approved by the ethics committee of the hospital (Yedikule Chest Diseases and Thoracic Surgery Training and Research Hospital).

One hundred consecutive patients with active lung tuberculosis referred to Chest Diseases and Thoracic Surgery Training and Research Hospital between 2008 and 2010 (Group 1), 50 age and gender matched patients with community-acquired pneumonia (CAP, Group 2) and 28 healthy controls (Group 3) were included in the study. Sputum AFB (with Ziehl-Neelsen stain) and at least one culture (Löwenstein-Jensen media and BACTEC TB 460 system) were found positive in all patients in the group 1 and accepted as active lung tuberculosis. Patients with PTB were classified as stage 1 (minimal/mild), stage 2 (moderate) and stage 3 (advanced) according to the extent of the disease on the chest radiographs [7,8]. Lesions were considered to be ‘minimal’ if they were of slight to moderate density without cavitation and involved a part of one or both lungs, the total extent being less than the volume of lung on one side which was present above the second chondrosternal junction. Moderate lesions were present in one or both lungs, but the total extent did not exceed the following limits: disseminated lesions of slight to moderate density which extended throughout the total volume of one lung, or the equivalent in both lungs; dense and confluent lesions which were limited in extent to one third of the volume of one lung; total diameter of cavitation, when present, was less than 4 cm. When the lesions were more extensive than these were recorded as ‘advanced’. PTB patients were divided into two groups based on thrombocyte counts and a trombocyte count higher than 400x10^3^/l was considered as thrombocytosis: 56 PTB patients with normal thrombocyte count, 44 PTB patients with reactive thrombocytosis. Reactive Thrombocytosis: it is meant that PLT count is higher than 400.000/μL. CAP (Group 2) was defined as the presence of a new pulmonary infiltrate on chest radiography that was cleared by antibiotic therapy at the time of diagnosis associated with at least one of the following: cough, fever and leukocytosis. Subjects were excluded from the study if there was a history of severe cardiovascular disease, significant liver or autoimmune disorders, neoplasm and immunosuppressive conditions.

Normal healthy individuals presenting for routine examination and with no complaint or known disease with a normal chest radiography were included in the control group. Full blood counts were carried out using “ABX Pentra 120 (Impedance&Optical, Minnesota, USA)”, serum levels of CRP (C-Reactive Protein) with “Olympus AU2700 Plus Beckman Coulter (Tokyo, Japan)” and ESR (Eritrocyte Sedimentation Rate) with “Eriline AR Linear (Barcelona, Spain)” devices in each of the three groups.

### Statistical analysis

All the statistical analyses were performed using SPSS 11,5 package software (SPSS Inc., Chicago, IL, USA). “OnewayAnova Analysis of Variance” test was used in inter-group comparisons and “Pearson and Spearman Correlation Tests” in assessment of the correlation between the numerical and categorical parameters. Comparison of the laboratory values in PTB group according to radiological stage and existence of thrombocytosis was performed using “Mann Whitney-*U* Test.” p <0.05 values were considered statistically significant. The best cut-off values of PLT and PCT in differentiation of tuberculosis and pneumonia was calculated with “ROC Curve Analysis”.

## Results

WBC, ESR, CRP, PLT and PCT values were significantly higher both in Group 1 and Group 2 than in Group 3. On comparison of Group 1 and Group 3; Hb values were lower, while PDW values were higher. On comparison of Group 1 and Group 2; WBC, ESR and CRP values were lower, while PLT and PCT values were higher in the Group 1 compared to Group 2 (p < 0.001). PLT was correlated with CRP and ESR values in the tuberculosis group (r = 0.57, r = 0.56, p < 0.001; respectively), while it was not correlated with CRP and ESR in the pneumonia group (r = 0.20, r = 0.26, p > 0.05, respectively). Characteristics of the groups and laboratory outcomes are given in Table 1.

**Table 1:** Characteristics and laboratory outcomes of the groups

	**Group 1**	**Group 2**	**Group 3**	**Group 1-2**	**Group 1-3**	**Group 2-3**
	**(n = 100)**	**(n = 50)**	**(n = 28)**	**p value**	**p value**	**p value**
Age	38.9 ± 15.3	37.5 ± 15.1	39.4 ± 13.0	0.81	0.72	0.36
Sex, F/M	19/81	10/40	10/18	0.08	0.06	0.052
Hb, g/dL	12.3 ± 1.4	12.8 ± 1.5	13.4 ± 1.0	0.12	0.002*	0.19
WBC, μL	8473 ± 2222	18194 ± 4046	7157 ± 1528	<0.0001*	0.002*	<0.0001*
PLT,x10^3^/μL	384.5 ± 122	304.4 ± 88.3	253.78 ±55	<0.0001*	<0.0001*	0.008*
PDW, %	14.7 ± 2.0	14.5 ± 2.3	13.5 ± 1.8	0.90	0.004*	0.14
MPV, fL	8.54 ±1.39	8.74 ± 0.71	8.65 ± 0.52	0.58	0.89	0.90
PCT, %	0.33 ±0.09	0.27 ± 0.05	0.21 ± 0.04	<0.0001*	<0.0001*	0.007*
ESR, mm/h	69.2 ± 25.5	85.5 ± 21.6	15.9 ± 3.5	<0.0001*	<0.0001*	<0.0001*
CRP, mg./L	6.3 ± 4.7	15.5 ± 7.2	0.4 ± 0.2	<0.0001*	<0.0001*	<0.0001*

*Significant value (p < 0.05).

Group1, Pulmonary Tuberculosis; Group2, Pneumonia; Group 3, Healthy control; F/M,Female/Male; Hb, Hemoglobin; WBC, White blood cell; PLT, Platelet; PDW, Platelet distribution width; MPV, Mean platelet volüme; PCT,: Plateletcrit; ESR,Eritrocyte sedimentation rate; CRP, C-reactive protein.

Patients with pulmonary tuberculosis were classified as stage 1 (n = 8), stage 2 (n = 72) and stage 3 (n = 20) according to the radiological extent. Stage 1 and 2 patients (n = 80) were compared with stage 3 (n = 20) patients. ESR, CRP, PLT and PCT values were higher in the patients with advanced stage (stage 3), while Hb was found lower in these patients. The increase in the platelet counts and PCT in advanced stage PTB was correlated both with the increase in CRP (r = 0.69, p < 0.01) and ESR (r = 0.56, p < 0.05). In addition, the rate of male gender was found higher in advanced stage tuberculosis. General characteristics and laboratory outcomes of the patients with pulmonary tuberculosis according to the radiological stage are given in Table 2.

**Table 2:** General characteristics and laboratory outcomes of the patients with pulmonary tuberculosis according to the radiological stage

	**Stage 1,2 PTB**	**Stage 3 PTB**	**p value**
	**(n = 80)**	**(n = 20)**	
Age	39.2 ± 15.9	37.8 ± 12.9	0.47
Sex, F/M	18/62	1/19	0.02*
Hb, g/dL	12.5 ± 1.5	11.9 ± 1.2	0.004*
WBC, μL	8369 ± 2398	8890 ±1266	0.85
PLT, x10^3^/μL	357 ± 108	491 ± 120	<0.0001*
PDW, %	14.8 ± 2.0	14.0 ± 2.2	0.23
MPV, fL	8.6 ± 1.5	8.3 ± 1.0	0.52
PCT, %	0.30 ± 0.07	0.4 ± 0.1	<0.0001*
ESR, mm/h	66 ± 26	83 ± 17	0.003*
CRP, mg/L	5.7 ± 4.9	9.1 ± 3.0	<0.0001*

*Significant value (p < 0.05).

PTB, Pulmonary tuberculosis; F/M, Female/Male; Hb, Hemoglobin; WBC, White blood cell; PLT, Platelet; PDW, Platelet distribution width; MPV, Mean platelet volüme; PCT, Plateletcrit; ESR, Eritrocyte sedimentation rate; CRP, C-reactive protein.

Patients with tuberculosis were divided into two groups as the patients with a normal platelet count (n = 56) and those with thrombocytosis (n = 44). WBC, ESR, CRP and PCT values were higher in the patients with thrombocytosis compared to those with a normal platelet count, while Hb was found lower in these patients. In addition, radiological advanced stage disease was more common in the patients with thrombocytosis. General characteristics and laboratory outcomes of the patients with pulmonary tuberculosis according to the platelet count are given in Table 3.

**Table 3:** General characteristics and laboratory outcomes of the patients in Group 1 (PTB) according to the platelet count

	**PTB (Thrombocytosis)**	**PTB (Normal Platelet Count)**	**P value**
	**(n = 44)**	**(n = 56)**	
Age	40.5 ± 15.4	37.7 ± 15.3	0.28
Sex, F/M	8/36	11/45	0.07
Stage 1,2/Stage 3	28/16	52/4	<0.0001*
Hb, g/dL	12 ±1.3	13 ± 1.3	<0.001*
PDW, %	14.9 ± 2.2	14.4 ± 1.9	0.32
PCT, %	0.39 ± 0.08	0.26 ± 0.06	<0.0001*
WBC, μL	9400 ± 2264	7745 ± 1912	<0.0001*
ESR, mm/h	85 ± 20	57 ± 23	<0.0001*
CRP, mg/L	9.2 ±4.4	4.1 ±3.7	<0.0001*

*Significant value (p < 0.05).

PTB, Pulmonary tuberculosis; Hb, Hemoglobin; PDW, Platelet distribution width; PCT,Plateletcrit; WBC, White blood cell; ESR,Eritrocyte sedimentation rate; CRP, C-reactive protein.

**Figure 1:**
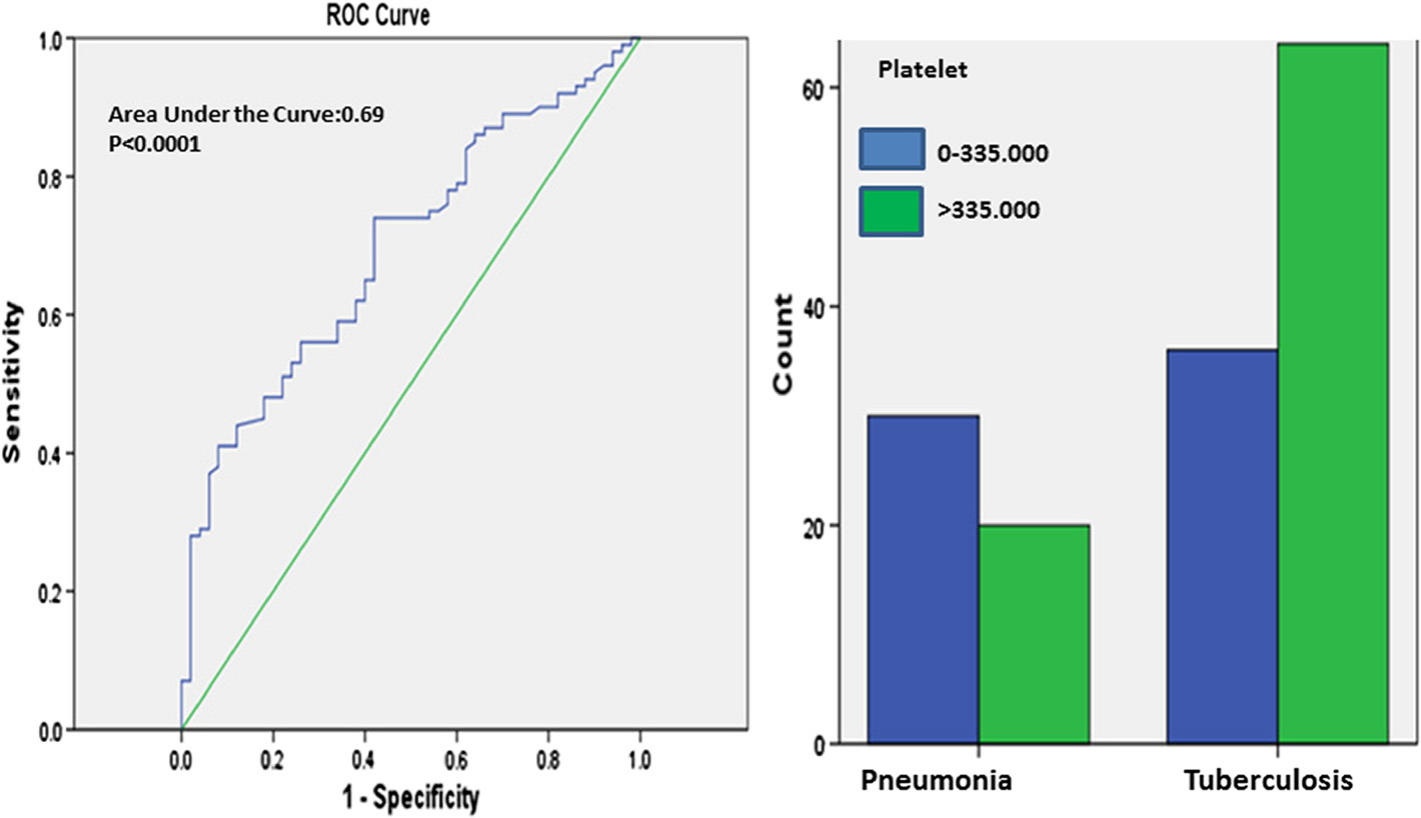
**ROC curve for using PLT levels in differential diagnosis of pulmonary tuberculosis and pneumonia.** Comparison of groups at the best cut-off point (335.000/μL).

ROC curve analysis was performed for the PLT and PCT values among TB patients vs controls (pneumonia and pneumonia + healthy control). The best PLT cut-off value in differential diagnosis of tuberculosis and pneumonia was defined as 335000/μL. For this cut off value, sensitivity was found as 64%, specificity as 60%, positive predictive value as 76.2% and negative predictive value as 45.5% (Figure 1).

**Figure 2:**
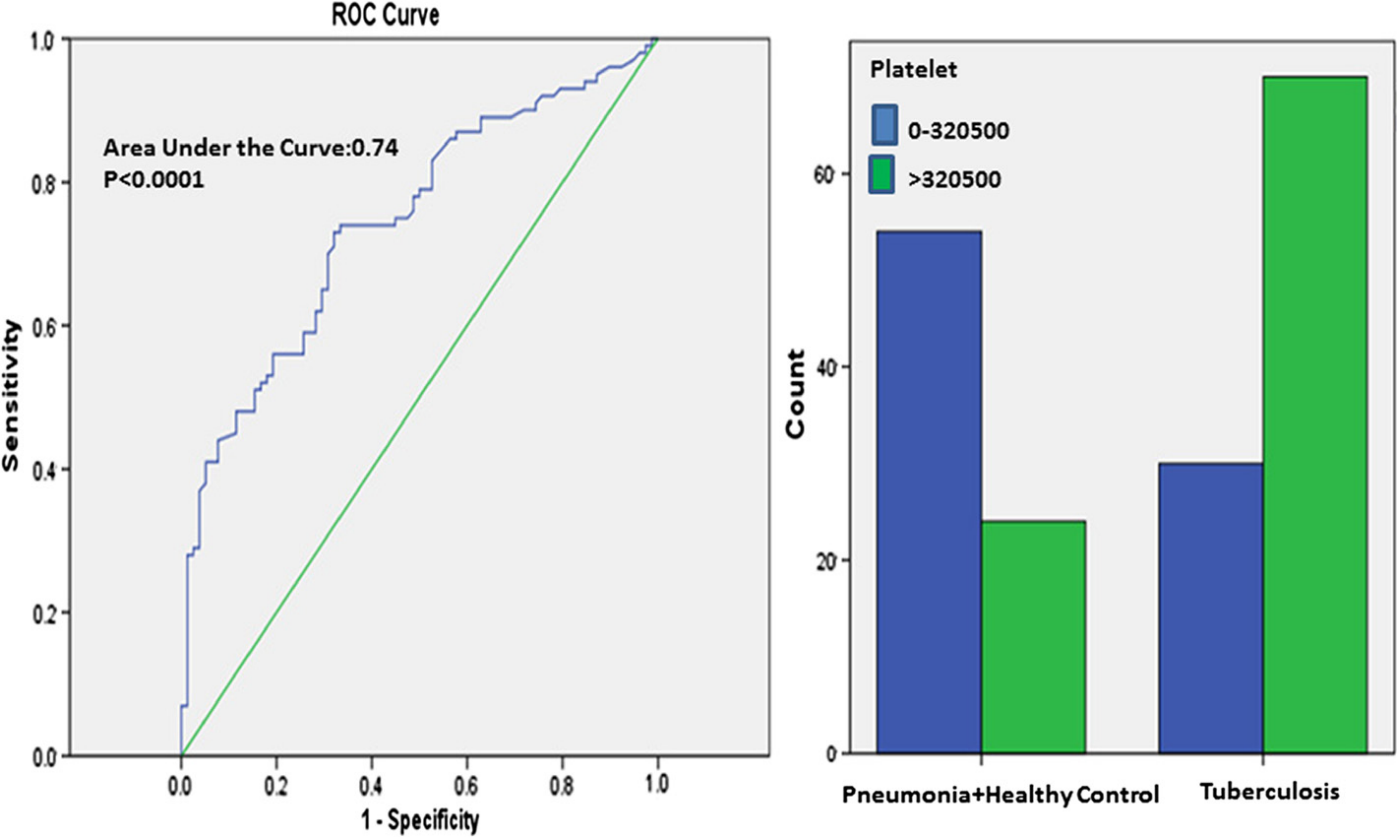
**ROC curve for using PLT levels in differential diagnosis of pulmonary tuberculosis and pneumonia + healthy control.** Comparison of groups at the best cut-off point (320.500/μL).

The best PLT cut-off value in differential diagnosis of tuberculosis and pneumonia + healthy control was defined as 320000/μL. For this cut off value, sensitivity was found as 70% specificity as 69.2%, positive predictive value as 74.5% and negative predictive value as 64.3% (Figure 2).

**Figure 3:**
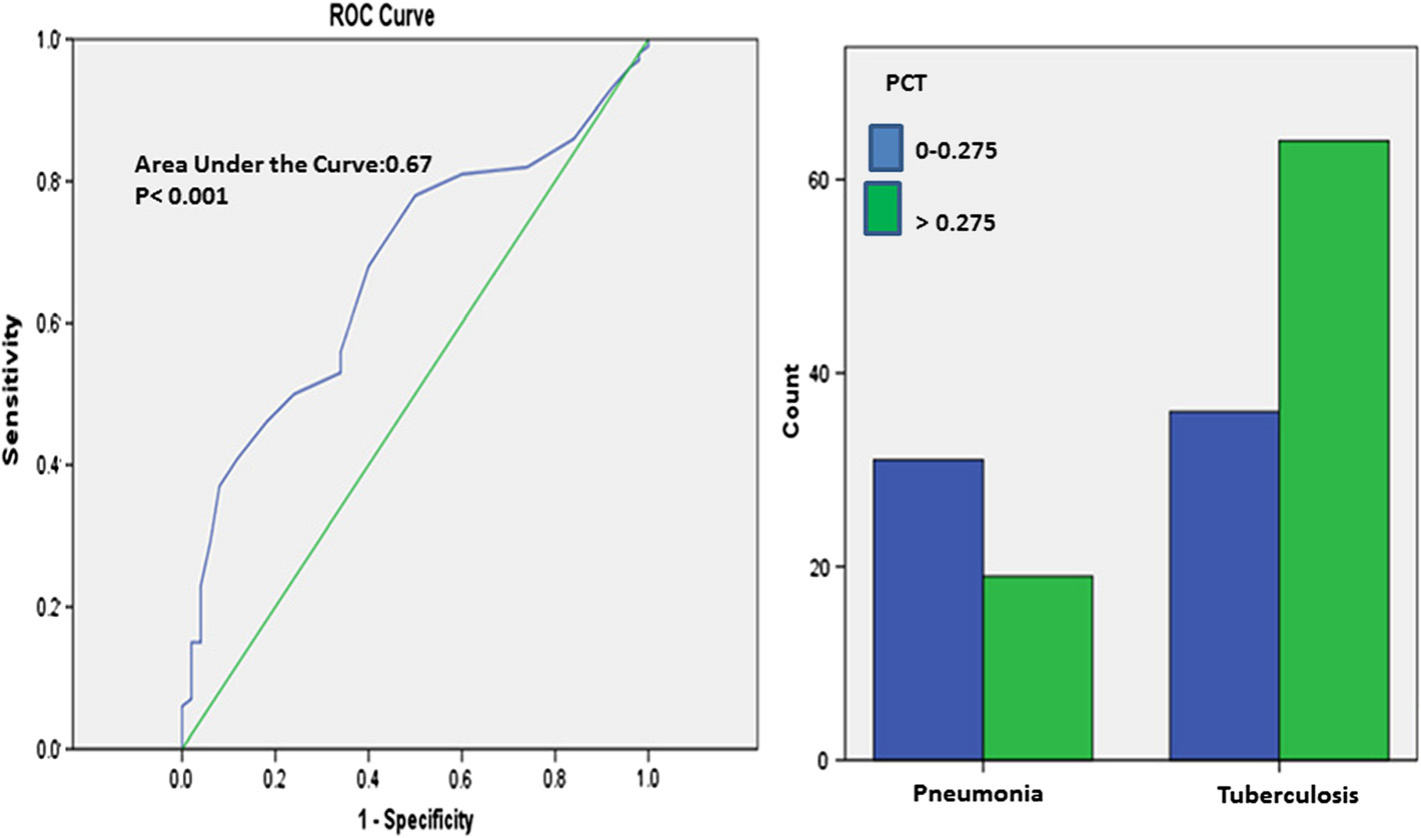
**ROC curve for using PCT levels in differential diagnosis of pulmonary tuberculosis and pneumonia.** Comparison of groups at the best cut-off point (0.275%).

The best PCT cut-off value in differential diagnosis of tuberculosis and pneumonia was defined as 0.275%. For this cut off value, sensitivity was found as 64%, specificity as 62%, positive predictive value as 77.1% and negative predictive value as 46.3% (Figure 3).

**Figure 4:**
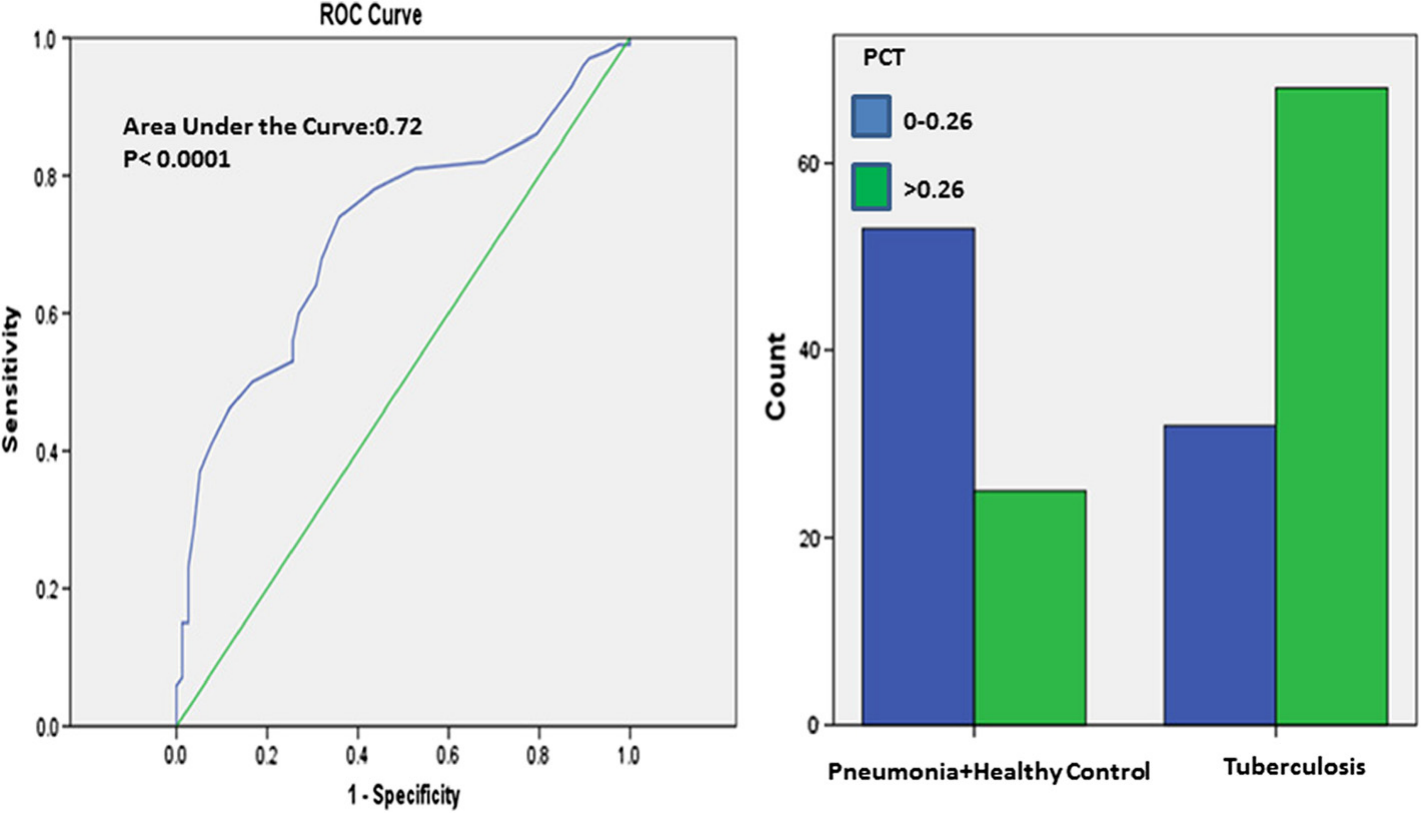
**ROC curve for using PCT levels in differential diagnosis of pulmonary tuberculosis and pneumonia + healthy control.** Comparison of groups at the best cut-off point (0.26%).

The best PCT cut-off value in differential diagnosis of tuberculosis and pneumonia + healthy control was defined as 0.26%. For this cut off value, sensitivity was found as 68%, specificity as 67.9%, positive predictive value as 73.1% and negative predictive value as 62.4% (Figure 4).

## Discussion

Reactive thrombocytosis developing in PTB has been shown to be correlated with acute phase reactants and severity of the disease [3,4,9]. In this study, we found thrombocytosis in 44% of PTB patients. The increase of the platelet counts in advanced stage PTB was correlated both with the increase in CRP and ESR. Referring to the indices of platelets, PCT was found higher in PTB than in pneumonia and control groups, while PDW was found increased only in PTB, not in healthy controls. PCT was accepted as an indicator of circulating platelets in a unit volume of blood [10]. Our results indicate that the increase in PCT is more distinctive and significant for tuberculosis than the other platelet indices. Tozkoparan et al. found platelet count and all indices (PDW, MPV and PCT) higher in active PTB than in pneumonia and inactive tuberculosis. They demonstrated that these values decrease to normal levels after the treatment. They pointed out that the increase in platelet count and indices might be used in addition to the other parameters in order to evaluate the activation of tuberculosis [1].

In earlier studies, multiple microthrombi have been found to occur around the tuberculosis cavity, and this has been thought to be a defense mechanism preventing the dissemination of tuberculosis [11]. Similarly, Khechinashvili et al. demonstrated with electron microscopy that the structure of the platelet changes in fibrocavernous tuberculosis and supposed that this structural change might be associated with the immunological process of tuberculosis [11,12]. In our study, PLT, PCT, WBC, CRP and ESR values were significantly higher both in PTB and pneumonia groups than in healthy controls. PLT and PCT values were higher, while CRP, ESR and WBC values were lower in PTB than in the pneumonia group. However, although the increase in PLT was correlated with acute phase reactants (CRP, ESR) in PTB, in pneumonia there was no correlation. These results suggest that platelets might play a different role in the inflammatory response developing against PTB.

In their study with PTB patients, Bozaky et al. demonstrated that hematological abnormalities are more common in severe tuberculosis. The most common of their findings were anemia, leukocytosis, leukopenia and thrombocytosis [5]. Again in a similar study from India, full blood count, bone marrow (BM) aspiration and BM biopsy were carried out in 32 patients with disseminated/miliary and 23 with pulmonary tuberculosis. The most common findings were thrombocytopenia and severe anemia in patients with disseminated/miliary and thrombocytosis in those with pulmonary tuberculosis [13]. Other studies also reported the most common findings in patients with pulmonary tuberculosis as anemia, leukocytosis, elevated ESR and thrombocytosis [4,14-17]. In our study, we found PLT, PCT, ESR and CRP values higher and Hb value lower in the advanced stage PTB compared to the other stages. Furthermore, the increase in PLT was correlated with the increase in CRP and ESR. Thus, we demonstrated the increase in platelet count and inflammatory response to be higher in advanced stage PTB, with a positive correlation between them. In line with our study, there are in the literature several investigations showing the acute phase response to be greater in the severe tuberculosis [1,18,19].

Ünsal et al. found levels of IL6 and CRP higher in PTB patients with thrombocytosis than in those with PTB and a normal platelet count. They pointed out the inflammatory response to be greater in PTB patients with thrombocytosis, and this is possibly associated with IL-6 [2]. Similarly,Baynes et al. showed that there is a significant correlation between the degree of thrombocytosis and that of inflammation (ESR and CRP values) in PTB [20]. In an *in vitro* study, platelet activation was evaluated according to the levels of platelet factor-4, and a correlation of good degree was shown between the platelet activation and radiological extent of tuberculosis [9]. In our study we also found ESR, CRP, WBC and PCT values to be higher, and Hb value lower, in PTB patients with thrombocytosis than in those with a normal platelet count. In addition, we encountered the disease with radiological advanced stage more frequently in the tuberculosis patients with thrombocytosis.

## Conclusions

Our study indicated that following different results compared to other studies:

WBC, ESR, CRP and PCT values were higher in the tuberculosis patients with thrombocytosis compared to those with a normal platelet count (p < 0.0001).

PDW was higher in the tuberculosis group than in healthy controls (p < 0.05), whereas there is no correlation between pneumonia group and healthy controls (p > 0.05).

PLT was correlated with CRP and ESR values in the tuberculosis group, while it was not in the pneumonia group.

Only PLT and PCT were correlated with radiological extent in the PTB group.

No significant difference was found in levels of MPV in patients with tuberculosis and pneumonia compared to healthy controls.

In summary, it is known that thrombocytosis is caused by chronic inflammation, and the extensive PTB disease is the result of a diagnostic delay (PTB history of several months). This study indicates that reactive thrombocytosis and higher PCT and PDW levels develop frequently in PTB and there is a strong correlation between thrombocytosis and acute phase reactants, that is the inflammatory response. In addition, tuberculosis with radiological advanced stage is seen more frequently in patients with thrombocytosis and higher PCT levels, thus drawing attention to a possible role of platelets in the immunopathogenesis of tuberculosis.

## **Competing interests**

The authors declare that they have no competing interests.

## **Authors’ contributions**

FS: Formulating the hypothesis, writing of the manuscript, data entry and data analysis; EY: Data entry and analysis, writing of the manuscript; PY: Review of the manuscript, data analysis. All authors read and approved the final manuscript.
